# The Effect of Qigong on Depressive and Anxiety Symptoms: A Systematic Review and Meta-Analysis of Randomized Controlled Trials

**DOI:** 10.1155/2013/716094

**Published:** 2013-05-20

**Authors:** Chong-Wen Wang, Cecilia Lai Wan Chan, Rainbow T. H. Ho, Hector W. H. Tsang, Celia Hoi Yan Chan, Siu-Man Ng

**Affiliations:** ^1^Centre on Behavioral Health, The University of Hong Kong, Hong Kong; ^2^Department of Social Work & Social Administration, The University of Hong Kong, Hong Kong; ^3^Department of Rehabilitation Sciences, The Hong Kong Polytechnic University, Hong Kong

## Abstract

*Objective*. To evaluate clinical trial evidence of the effectiveness of qigong exercise on depressive and anxiety symptoms. *Methods*. Thirteen databases were searched from their respective inception through December 2012. Relevant randomized controlled trials (RCTs) were included. Effects of qigong across trials were pooled. Standardized mean differences (SMDs) were calculated for the pooled effects. Heterogeneity was assessed using the *I* ^2^ test. Study quality was evaluated using the Wayne Checklist. *Results*. Twelve RCTs met the inclusion criteria. The results of meta-analyses suggested a beneficial effect of qigong exercise on depressive symptoms when compared to waiting-list controls or usual care only (SMD = −0.75; 95% CI, −1.44 to −0.06), group newspaper reading (SMD = −1.24; 95% CI, −1.64 to −0.84), and walking or conventional exercise (SMD = −0.52; 95% CI, −0.85 to −0.19), which might be comparable to that of cognitive-behavioral therapy (*P* = 0.54). Available evidence did not suggest a beneficial effect of qigong exercise on anxiety symptoms. *Conclusion*. Qigong may be potentially beneficial for management of depressive symptoms, but the results should be interpreted with caution due to the limited number of RCTs and associated methodological weaknesses. Further rigorously designed RCTs are warranted.

## 1. Introduction

Mental disorders are prevalent among the general population. It is estimated that 26.2% of Americans aged 18 and older suffer from a diagnosable mental disorder in a given year [1], and that about one in five adults in China has a current mental disorder (1-month prevalence 17.5%) [2]. Mental disorders are reported to be more debilitating than most chronic physical conditions [3].

Depression and anxiety disorders are common mental disorders worldwide, which are significantly associated with morbidity, disability, comorbidities, and mortality [4]. According to World Health Organization (WHO), depression is ranked as the 4th global burden of disease; it is projected that it will be the 2nd most important cause of disability (after heart disease) and the second-leading cause of disease burden worldwide by the year 2020 [5]. Because of traditional beliefs and attitudes to mental problems among general population in many societies, patients with depression and anxiety disorders may confront dilemmas and experience stigma, which discourage them from seeking medical treatment and psychological therapy. A recent survey in England suggested that only a quarter of all those with mental problems were in treatment [3]. Moreover, existing evidence suggests that the efficacy and effectiveness of currently available antidepressants and psychotherapy seem unacceptably low [6]. Furthermore, antidepressants may cause side effects in various organic systems, making them unacceptable by some patients [7]. Thus, seeking alternative and complementary therapies for management of their symptoms is preferred by many patients with depressive and anxiety disorders or symptoms.

Qigong, a modality of traditional Chinese medicine (TCM), is originally a form of ancient martial arts that has been developed and used to improve physical fitness and strength in China for thousands of years [8]. The basic components of qigong include concentration, relaxation, meditation, breathing regulation, body posture, and movement [8]. According to TCM philosophy, qigong aims to achieve a harmonious flow of vital energy (*qi*) in the body and regulate the functional activities of the body through regulated breathing, mindful meditation, and gentle movements. With regular practice and rehearsal of the structured movements as well as the atonement of mind and breath, practitioners may experience greater improvement in strength and fitness. Long-term practice of qigong may help to prevent illness, maintain good health, and heal the body from diseases. Basically, there are two types of qigong: internal qigong and external qigong. Internal qigong or qigong exercise is self-directed and involves the use of movements, meditation, and controlled breathing pattern, whereas external qigong is usually performed by experienced masters using their hands to direct *qi* energy (emitted *qi*) onto the patient for healing or treatment [9–11]. Although there is little scientific evidence to support the emitted “*qi*” and its therapeutic effects [12], qigong exercise is popularly practiced by a large number of people in Chinese communities to improve their health. Many styles of qigong, such as “The Five-Animal Play (Wuqinxi),” “The Eight-Section Brocades (Baduanjin),” and “Guolin Qigong,” have been developed. Generally, qigong can be classified into two categories: dynamic qigong (*dong gong*) and static qigong (*jing gong*). The former involves the coordination of movements and meditation, whereas the later focuses on mind concentration and body relaxation without physical movement [8]. Qigong is an easily adaptable form of mind-body integrative exercise that can be practiced in anyplace, and anytime, without any special equipment. It is widely practiced by Chinese not only to improve their physical health [9–11, 13–15], but also to control their emotions, manage their stress or depressive/anxiety symptoms, and enhance overall well-being.

In recent years, an increased number of studies have documented the effect of qigong on depressive and anxiety symptoms. The mechanisms underlying the possible antidepressive effect of qigong have also been speculated upon [16–18]. However, clinical trial evidence on health benefits of qigong for patients with depressive and anxiety disorders or symptoms has not been critically examined yet. Although two relevant systematic reviews [19, 20] have been published most recently, their conclusions are inconsistent and subject to bias because of methodological flaws. In one review [19], the great heterogeneity of participants (including healthy subjects, subjects with chronic illnesses, and subjects with depression) and a wide spectrum of outcomes (including mood, anxiety, psychological well-being, self-efficacy, and quality of life) included in that review, coupled with selective meta-analysis of only three of the fifteen included studies, undermined the reliability of its conclusive statements. In another review [20], the conclusive statements were rather evasive due to a great diversity of study populations (including college students and patients with cancer, severe chronic pain, Parkinson's disease, or other chronic illnesses) examined in the review and failure to perform meta-analysis of the results of the included trials. In light of the limitations of the previous reviews, the purpose of the present systematic review was to critically evaluate the overall effectiveness of qigong as a form of mind-body integrative exercise on depressive and anxiety symptoms using standardized inclusion criteria for each of the PICO (participants, interventions, comparisons, and outcomes) elements as detailed in the Cochrane Handbook [21].

## 2. Methods

### 2.1. The Literature Search

The following electronic databases were searched from their respective inception through December 2012: PubMed/MEDLINE, CENTRAL, CINAHL, EMBASE, AMED, Qigong and Energy Medicine Database, China Academic Journals Full-Text Database, Medicine/Hygiene Series, China Proceedings of Conference Full-Text Database, China Master's Theses Full-Text Database, China Doctoral Dissertations Full-Text Database, Taiwan Electronic Theses and Dissertation System, Taiwan Electronic Periodical Services, and Index to Taiwan Periodical Literature System. The search terms used for this systemic review include *qigong, qi-gong, qi gong, chi chung, chi gong, qi chung, qi-training, depression, depressive, depressed, dysthymia, dysthymic, anxiety, burnout, mental*, and *psychiatric*. Both traditional and simplified Chinese translations of these terms were used in Chinese databases. Reference lists of all included studies, relevant reviews, and other archives of the located publications were hand-searched for further relevant articles.

### 2.2. Study Selection

The following criteria were applied for study selection.

*(1) Types of Studies*. All RCTs aiming to examine the effects of qigong on depressive and anxiety symptoms were included. Nonrandomized controlled clinical trials (CCTs) were excluded due to their susceptibility to bias. Noncontrolled observational studies and case reports were excluded due to lack of significant evidence. 

*(2) Types of Participants*. Adult men and women aged 18 and over (with no upper age limit), who were defined by the authors of the trial as having depression and anxiety disorders or elevated depressive symptoms. Given the limited number of studies conducted among patients with primary depression or anxiety disorders, studies of qigong among patients with depressive/anxiety symptoms secondary to chronic illnesses were included, but the studies among patients with severe or specific conditions (e.g., cancer, asthma, congestive heart failure, Parkinson's disease, fibromyalgia, and severe chronic pain) were excluded. Studies among individuals with burnout syndrome or insomnia were also included if depressive symptoms were measured in the trial. Studies among healthy subjects, children, and pregnant women were excluded. 

*(3) Types of Intervention*. Studies comparing any style of qigong with waitlist/other forms of exercise/other types of intervention (e.g., psychotherapy) or studies comparing qigong plus another intervention versus the other intervention alone were included. Studies comparing qigong plus another intervention versus qigong alone were excluded. Studies that measured outcomes immediately before and after a single qigong session (immediate effects of qigong) were not included due to high risk for biases. 

*(4) Types of Outcome Measures*. The focuses of this review were on depressive and anxiety symptoms. Studies including outcome measures of depressive and/or anxiety symptoms either as continuous measures or as dichotomous outcomes were included. Studies focusing mood status, perceived stress, or other normal psychological reactions were excluded. Studies in which depressive and anxiety symptoms were measured with validated instruments that were designed to assess the severity of depressive and anxiety symptoms or to screen depressive and anxiety disorders were included. Studies in which depressive and anxiety symptoms were measured merely with nonspecific instruments that were not designed to screen depressive and anxiety disorders or to assess the severity of depressive and anxiety symptoms specifically were excluded. Studies focusing on other self-report outcomes (such as sleep quality) or biomarkers (such as cortisol) were not included if depressive and/or anxiety symptoms were not measured in the trials.

### 2.3. Data Extraction and Quality Assessment

For each included study, data were extracted from the original paper independently by one main researcher and then verified by another researcher. Data extracted included quality criteria, participants, quality and dosage of intervention, outcome measures, and results. Any discrepancies were discussed until consensus was achieved. The methodological quality of each trial was evaluated using the Wayne Checklist [22]. It was commended that this checklist was comprehensive, adaptable to clinical context, and compatible with recent developments in statistics and experimental design [23]. The checklist assesses study quality with respect to reporting of the following criteria: randomization, details of randomization methods, clear inclusion and exclusion criteria, blinding of outcome assessors, description of withdrawal and dropouts, sample size estimates or justification, use of appropriate statistical analyses, details of qigong intervention, and qualification of the qigong instructors. Since blinding both investigators and participants were generally impossible for studies of qigong, the checklist only assessed if the outcome assessors were blind to treatment allocation. A trail was considered to have used intention-to-treat analysis if all the participants were analyzed with no difference in number between pre- and postintervention. The risk of bias in the included studies was assessed using the framework for methodological quality recommended by Jüni and colleagues [24]. According to this framework, biases fall into four categories: selection bias (biased allocation to comparison groups), performance bias (unequal provision of care apart from intervention under evaluation), detection bias (biased assessment of outcomes), and attrition bias (biased occurrence of loss to followup).

### 2.4. Data Synthesis and Analysis

Meta-analyses of the results reported in the included studies were performed using Review Manager 5.2 (http://ims.cochrane.org/revman). Effect sizes were calculated for each trial using Hedge's *g* [25]. Standardized mean differences (SMDs) were calculated for the pooled effects. We interpreted the SMDs using the following “rule of thumb”: 0.2 represents a small effect, 0.5 a moderate effect, and 0.8 a large effect [26]. A random-effects model was used for data synthesis when an outcome was measured by different measures, and a fixed-effects model was used when the outcome was measured by the same instrument. The *χ* ^2^ statistic, together with the *I* ^2^ statistic, was used to assess heterogeneity. Studies with an *I* ^2^ statistic of >75% were considered to have a high degree of heterogeneity, those with an *I* ^2^ statistic of 50%–75% were considered to have a moderate degree of heterogeneity, and those with an *I* ^2^ statistic of 25%–50% were considered to have a low degree of heterogeneity [27]. Sensitivity analyses were conducted by omitting one study in turn and evaluating the influence of a single study on the overall pooled effect. Publication bias was not examined due to the limited number of studies (<10) included in each analysis. A value of *P* < 0.05 was considered statistically significant. 

Where an outcome was assessed by more than one tool in a trial, we included the main outcome measure (identified as the first outcome reported in the results section or the outcome reported in the abstract) only in the meta-analysis. For publications in which only *P* values were reported but the data on continuous outcome measures were not available, we wrote to the corresponding authors for relevant data. Trials using dichotomous data as primary outcomes were not included in our meta-analyses. Trials presenting median and interquartile range (indicators of skewed data) rather than mean and standard deviation were also not included in our meta-analyses due to impossibility of transformation between them. Where trials had more than two arms (e.g., qigong, other exercises, or waiting-list), we used data from the exercise arm for two separate comparisons: qigong versus other exercises and qigong versus waiting-list control. 

## 3. Results

### 3.1. Results of the Literature Search

**Figure 1:**
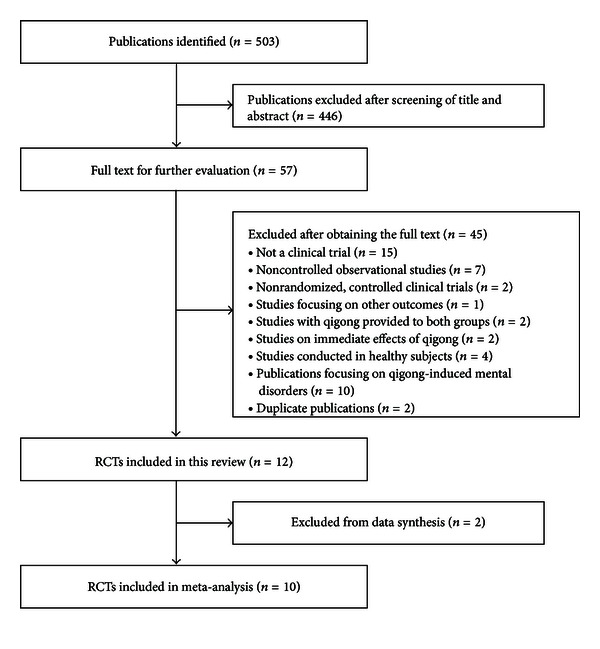
Selection process for included studies.

Our database searches identified 503 potentially relevant articles, of which 446 articles were excluded after screening of title or abstract. Full reports of 57 studies were acquired and 45 were further excluded due to that they were (1) not a clinical trial, (2) uncontrolled observational studies, (3) nonrandomized, controlled clinical trial, (4) studies comparing qigong plus another intervention versus qigong alone, (5) studies focusing on other outcomes, (6) studies on acute effects of qigong, (7) studies conducted in healthy subjects, (8) publications focusing on qigong-induced mental disorders, and (9) duplicate publications (Figure 1).

### 3.2. Description of Included Studies

Twelve RCTs [28–39] met our inclusion criteria. These studies were conducted in Hong Kong [28–30, 35–37], Sweden [34], and Mainland China [31–33, 38, 39], respectively. Ten of them were published in peer-review journals with full texts and the remaining two were unpublished master theses [33, 39]. Eight RCTs were published in English and two [31, 32] were published in Chinese. The characteristics of the included studies are shown in Table 1. 

Participants in the included studies included patients with clinical depression [28, 33], depressed elders with chronic illnesses [35–37], patients with burnout syndrome [34], adults with depressive mood [29], women with perimenopausal syndrome and depression [32], and patients with depressive symptoms secondary to chronic conditions including hypertension [30], diabetes mellitus [31, 39], and subhealth status [38]. Sample sizes in the included studies ranged from 38 to 145 with a total of 936 participants including 428 subjects in the qigong groups and 508 subjects in control groups. 

Qigong exercise used in the included studies included the Eight-Section Brocades (Baduanjin) [31, 32, 35–37, 39], Wuqinxi [33, 38], Guolin Qigong [30], and Dejian mind-body intervention based on traditional *Shaolin* qigong practice [28, 29]. The style of qigong was not mentioned in one study [34]. Duration of qigong intervention ranged from 4 weeks [29] to 16 weeks [30, 36]. All included studies just only examined the effect of qigong immediately following the qigong intervention, and no study had examined the effect of qigong intervention after a period of followup. 

A two-armed, parallel group design was employed in nine studies, in which qigong was compared with newspaper reading and discussion [35, 36], cognitive-behavioral therapy [29], walking [32, 33] or conventional exercise [30], usual care or treatment [31, 34, 36], and waiting-list controls [28, 38, 39]. Three trials were conducted with a three-armed, parallel group design. In one trial qigong was compared with cognitive-behavioral therapy and waiting-list controls [28]. In another trial qigong group was compared with a walking group and a waiting-list control group [32]. In the last trial qigong group was compared with a mindful relaxation group and a waiting-list control group [39]. 

Regarding outcome measures, depressive symptoms were assessed in all of the included studies, whereas anxiety symptoms were assessed only in four studies. The depression scales applied in the examined studies included Geriatric Depression Scale [35–37], Beck Depression Inventory [28–30], Hospital Anxiety and Depression Scale [34], Hamilton Rating Scale for Depression [28, 36], Hamilton Depression Rating Scale [33], Self-rated Depression Scale [31, 38, 39], and Center for Epidemiologic Studies Depression Scale [32]. Anxiety scales applied in these studies included Beck Anxiety Inventory [30], Hospital Anxiety and Depression Scale [34], and Self-rated Anxiety Scale [38, 39]. 

### 3.3. Effects of Qigong on Depression or Symptoms

Of the included 12 studies, nine suggested a favorable effect of qigong on depressive symptoms [28, 29, 31–33, 35, 36, 38, 39] and three did not [30, 34, 37]. Because of heterogeneity of controls across the included studies, it would be inappropriate to synthesize the results of these studies directly, and so the effects of qigong on depressive symptoms were pooled by types of controls in this review. Ten trials were included in our meta-analyses. Two trials could not be included. In one trial, the values of median and interquartile range, rather than mean and standard deviation, were reported for outcome measures [34]. In another trial, only mean differences were presented [39].

**Figure 2:**
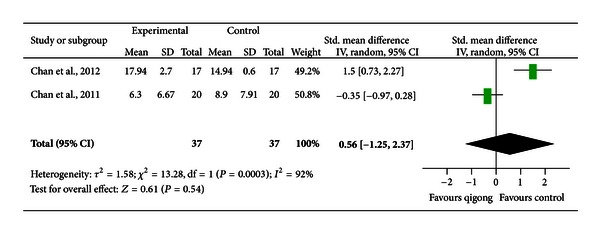
A forest plot of the meta-analysis of two studies comparing qigong to cognitive-behavioral therapy for changes in depressive symptoms.

*Comparison 1: Qigong versus Cognitive-Behavioral Therapy (CBT).* Two trials [28, 29] compared the effect of qigong on depressive symptoms with that of cognitive-behavioral therapy. One trial [28] focused on outpatients with clinical depression and another [29] focused on adults with depressive mood. Both trials suggested no intergroup difference. Their results were pooled and the pooled SMD was 0.56 [−1.25, 2.37], indicating an insignificant effect (*P* = 0.54; Figure 2). There was a high degree of heterogeneity (*I* ^2^ = 92%).

**Figure 3:**
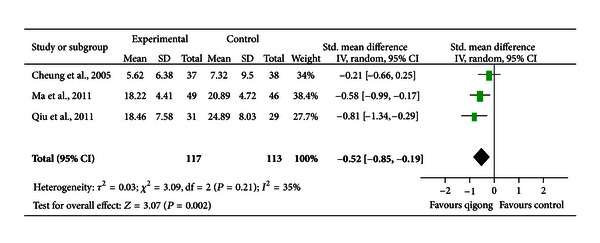
A forest plot of the meta-analysis of three studies comparing qigong to walking or conventional exercise for changes in depressive symptoms.

*Comparison 2: Qigong versus Walking or Conventional Exercise.* Three trials compared the effect of qigong with that of walking and conventional exercise [30, 32, 33]. Participants in these studies included women with perimenopausal syndrome and depression [32], patients with hypertension and depressive symptoms [30], and patients with mild or moderate depression [33]. Two studies suggested a beneficial effect of qigong [32, 33] and the remaining one did not [30]. Their results were pooled and the pooled SMD was −0.52 [−0.85, −0.19], indicating a moderate effect (*P* < 0.01; Figure 3). A low degree of heterogeneity was indicated (*I* ^2^ = 35%). Exclusion of the trial conducted by Cheung et al. [30] did not alter the pooled effect but resolved heterogeneity (SMD = −0.67 [−0.99, −0.34]; *P* < 0.001; *I* ^2^ = 0%). Exclusion of the trial conducted by Qiu et al. [33] mildly altered the pooled effect (SMD = −0.41 [−0.77, −0.04]; *P* < 0.05; *I* ^2^ = 30%). Exclusion of the trial conducted by Ma et al. [32] altered the pooled effect significantly (SMD = −0.49 [−1.09, −0.10]; *P* > 0.05; *I* ^2^ = 66%).

**Figure 4:**
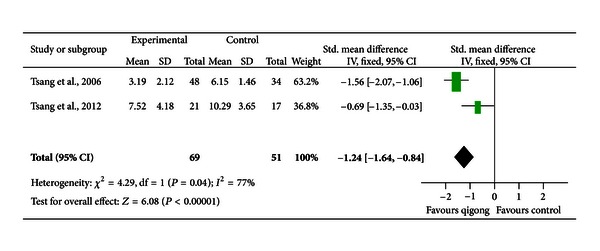
A forest plot of the meta-analysis of two studies comparing qigong to group newspaper reading for changes in depressive symptoms.

*Comparison 3: Qigong versus Group News Reading*. The effect of group support on depressive symptoms was adjusted in two trials [35, 36], in which group qigong training was compared to group newspaper reading. Both studies suggested a significant intergroup difference in favor of qigong. The pooled SMD was −1.24 [−1.64, 0.84], indicating a large effect (*P* < 0.001; Figure 4). There was a high degree of heterogeneity (*I* ^2^ = 77%).

**Figure 5:**
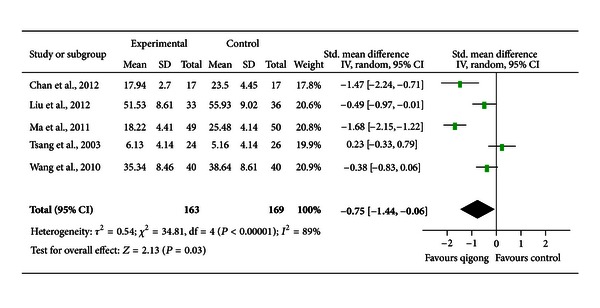
A forest plot of the meta-analysis of five studies comparing qigong to waiting list controls or qigong plus usual care to usual care only for changes in depressive symptoms.

*Comparison 4: Qigong or Qigong Plus Usual Care versus Waiting-List Controls or Usual Care Only.* Four trials [28, 32, 38, 39] compared qigong to waiting list controls and three trials [31, 34, 37] compared qigong plus usual care to usual care only. As mentioned before, two trials [34, 39] could not be included for meta-analysis. One reported negative results [34], whereas another suggested positive results [39]. Of the remaining five studies that could be included for meta-analysis, four trails [28, 31, 32, 38] suggested a favorable effect of qigong on depressive symptoms and only one trial [37] did not. Their results were pooled and the pooled SMD was −0.75 [−1.44, −0.06], indicating a moderate effect (*P* = 0.03, Figure 5). There was a high degree of heterogeneity (*I* ^2^ = 89%). Exclusion of the trial conducted by Chan et al. (2012) [28] significantly altered the pooled effect (SMD = −0.59 [−1.36, 0.18]; *P* = 0.13; *I* ^2^ = 90%). Exclusion of the trial conducted by Liu et al. (2012) [31] mildly altered the effect (SMD = −0.82 [−1.72, 0.08]; *P* = 0.07; *I* ^2^ = 91%). Exclusion of the trial conducted by Ma et al. (2011) [32] also altered the effect (SMD = −0.48 [−1.04, 0.08]; *P* = 0.09; *I* ^2^ = 76%). Exclusion of the trial conducted by Tsang et al. (2003) [37] increased the pooled effect (SMD = −0.99 [−1.68, −0.30], *P* < 0.01; *I* ^2^ = 86%). Exclusion of the trial conducted by Wang et al. (2010) [38] altered the pooled effect mildly (SMD = −0.85 [−1.74, −0.04], *P* = 0.06; *I* ^2^ = 91%).

### 3.4. Effects of Qigong on Anxiety Symptoms

The effect of qigong on anxiety symptoms was examined in four studies, in which participants included patients with burnout syndrome [34], subjects with sub-health status [38], patients with hypertension [30], and patients with diabetes mellitus [39]. Only one [38] of them suggested a significant difference in the effects of qigong on anxiety symptoms between the qigong intervention groups and the control groups, whereas the other trials [30, 34, 39] suggested otherwise.

### 3.5. Study Quality Assessment

Quality assessment for each trial was presented in Table 2. As shown in the table, inclusion and exclusion criteria were clearly defined in ten trials [28–36, 39]. Randomization method was reported in seven studies [29–31, 33–35, 39]. Allocation concealment was adequate only in five studies [28, 30, 35, 37, 39]. Blinding of outcome assessors was applied only to four studies [28, 29, 35, 36]. The number of those who did not complete the intervention program and thus did not provide data of postintervention assessment was reported in seven studies [28, 30, 31, 34–36, 39], of which intention-to-treat analyses were performed only in four trials [28, 30, 34, 35]. For remaining five studies [29, 32, 33, 37, 38] in which the rate of dropout was not reported and intention-to-treat analysis was not specified, we assumed that all participants in those studies have completed the intervention program. Sample size estimation was calculated or justified appropriately only in three studies [30, 34, 36].

## 4. Discussion

In this review, clinical trial evidence of the effectiveness of qigong on depressive and anxiety symptoms was comprehensively and critically examined. The strengths of the current review include the use of a systematic and transparent literature search, standardized inclusion criteria based on PICO, and careful meta-analyses of the results reported in the included studies by types of controls. On the basis of available evidence, our review demonstrated a beneficial effect of qigong on depressive symptoms when compared to waiting-list controls or usual care alone (SMD = −0.75; 95% CI, −1.44 to −0.06), group newspaper reading (SMD = −1.24; 95% CI, −1.64 to −0.84), and walking (SMD = −0.52; 95% CI, −0.85 to −0.19), which might be comparable to that of cognitive-behavioral therapy (*P* = 0.54). These findings are in alignment with a systematic review of physical exercise for depression [40]. However, interpretation and generalization of our results should be treated with caution due to the limited number of the included studies in each comparison and the methodological problems inherent in these studies.

Clinically, there are a number of subtypes of depression. Usually, depression can be classified into primary depression and secondary depression [41]. Depression can also be classified into major depression, minor depression (sub-threshold depression), and chronic depression including dysthymic disorder [41–43]. In addition, a large number of individuals may have elevated depressive symptoms that do not meet the diagnosis criteria for clinical depression. Onset or development of depression may be induced by a number of risk factors. It is suggested that approximately one-third of the risk is inherited or genetic-related and two-thirds is environmental [6]. Generally, primary depression or major depression disorder may be induced by the interaction of genetic and environmental factors, whereas secondary depression, minor depression, and elevated depressive symptoms are more likely to be induced by environmental factors such as prolonged stress, chronic illnesses, or adverse life events.

Currently, studies of qigong on primary depression and anxiety disorders are still rare. Among the studies included in this review, only two RCTs [28, 33] focused on patients with clinical depression as a primary mental problem. Although a desirable effect of qigong on depressive symptoms was observed in the two RCTs, the finding may be biased since all participants were prescribed with antidepressants. Thus, it was difficult to segregate the effects of qigong from the effect of medication [28]. Another RCT [37] focusing on patients with diagnosed burnout syndrome suggested no significant effect of qigong on depressive symptoms. Two additional RCTs [35, 36] among older adults with clinical depression also demonstrated a beneficial effect of qigong on depressive symptoms, but the results could not be generalized to all patients with depressive disorders for that all participants in the two studies were older adults whose depressive symptoms were secondary to chronic illnesses. While major depression can develop at any age, the median age at onset is 32 [44]. It has been reported that major depression is the leading cause of disability in the US for ages 15–44 [5]. To date, clinical trials of qigong particularly focusing on young adults with major depression are still limited. In the absence of RCTs in the field, nonrandomized CCTs may provide alternative evidence. Two relevant, small-scale CCTs [45, 46] could be identified and a mildly significant effect of qigong on depressive and anxiety symptoms was demonstrated in the two trials. Unfortunately, such data are highly susceptible to bias and hence, provide, little scientific evidence. 

Conducting research of qigong among patients with primary depression faces many challenges. A major challenge may be the difficulty in recruiting sufficient participants into the trial, since many patients with depression are unwilling to participate in such type of informed group training due to perceived social stigma and concern for personal privacy. Instead, many studies examined in this review were conducted among patients with depressive symptoms secondary to chronic illnesses. 

Although a beneficial effect of qigong on depressive symptoms among patients with chronic illnesses was observed in most of the studies examined in this review, the result may not be generalized to patients with severe physical problems. For instance, one RCT [47] and two CCTs [48, 49] suggested no significant effect of qigong on depressive symptoms among cancer patients. Another RCT [50] also suggested no beneficial effect of qigong on depressive symptoms among patients with Parkinson's disease. However, a recent study [51] suggested a beneficial effect of qigong on depressive symptoms in women with breast cancer. Thus, the efficacy and effectiveness of qigong on depressive symptoms for patients with different physical problems should be further tested in future studies.

It should also be noted that a high risk of bias might have existed in the included studies due to some inherent methodological weaknesses. In most of them, qigong was preferentially provided to the intervention groups as a group therapeutic modality, whereas the control groups did not have a matched number of social contact hours with coparticipants. Thus, performance bias might have existed in these trials and a placebo effect might have occurred in patients who benefited from participation in group activities and the contact with other persons. Blinding outcome assessors for group allocation was unclear in 8 RCTs [30–34, 37–39], and selection bias might have been introduced in these trials. The proportion of dropouts and withdrawals were recorded in 7 RCTs [28, 30, 31, 34–36, 39]. Attrition bias might have been introduced in these trails. Of them, intention-to-treat analysis was not applied to three RCTs [31, 36, 39]. The probability of bias for the results might exist in these studies. In addition, sample size was not appropriately justified in 9 RCTs [28, 29, 31–33, 35, 37–39]. It is unclear if the sample sizes in some studies were large enough to avoid type II error. Lastly, depressive and anxiety symptoms were only measured before and immediately after the qigong intervention programs in all of the included studies. None of them had followed the participants up for a period of time after the intervention. Thus, there was little evidence of the long-term effect of qigong on depressive symptoms. 

An examination of the included studies also reveals a great disparity in the dosage and intensity of qigong exercise across the studies examined, which may make it difficult to compare the results of these studies. As shown in Table 1, there were many forms of qigong applied in the included studies, with different duration of group practice and recommendations for individual home practice. The frequency of qigong practice at home was reported in some studies but not in others. Most importantly, the amount of home practice of qigong was not measured nearly in all of those studies in which group practice of qigong was not applied daily. Given that some studies have suggested a relationship between amount of qigong practice and health outcomes [52], a beneficial effect might have not been observed in those participants with insufficient dosage and intensity of qigong practice. Therefore, the dosage or intensity of qigong exercise, including individual home practice, should be measured for each participant and taken into account in data analysis in future studies.

Although no adverse effects of qigong were reported in the included studies, it should be noted that qigong-induced mental disorders were documented in some other publications [53, 54]. It was suggested that qigong-induced mental disorders or “qigong-precipitated psychoses” were resulted from “unrealistic expectation of acquiring supernormal powers” or “inappropriate practice of qigong” [54]. Thus, it is not recommended to use qigong intervention for management of depressive symptoms among patients with severe mental disorders, especially younger patients for high risk of unrealistic expectations. Indeed, we have tried to locate all controlled trials of qigong on all types of mental disorders or disturbances but found that studies of qigong on other types of mental disorders or disturbances rather than depressive and anxiety disorders were rare. 

Our review has some limitations. The first one may be the potential incompleteness of the evidence reviewed, a common concern for any systematic reviews. The second one may be that we have not contacted with relevant authors to identify unpublished or ongoing studies. Thus, publication bias might have existed in the included studies and the effect sizes of qigong might have been overestimated since positive trials are more likely to be published than negative trials. In addition, we could not examine the effects of dosage and intensity of qigong and also could not differentiate the effects of dynamic and static qigong on depressive symptoms due to the limited number of the included studies. Moreover, we could not examine the effects of qigong on other outcomes such as quality of life for patients with mental health problems due to that the focus of the current systematic review was on depressive and anxiety symptoms. These issues should be addressed in future studies. 

## 5. Conclusion 

To summarize, this review shows that there is only preliminary evidence that qigong may be potentially beneficial for management of depressive symptoms. However, the results should be interpreted with caution due to limited number of studies and associated methodological weaknesses. The results of this review may not be applicable to patients with primary depression since most of the included studies were conducted among patients with secondary depression, minor depression, or elevated depressive symptoms. The results may also not be applicable to patients with depression secondary to adverse life events since most of the included studies were conducted among patients with chronic illnesses. The results may not be generalized to young adults with mental health problems since few studies were conducted particularly among this group of clients. Moreover, it seems that qigong is ineffective in relieving depressive symptoms for patients with severe physical problems such as cancer and Parkinson's disease. This review cannot make any recommendations of the effectiveness and the dosage of qigong for patients with depression. Particularly, it should be cautious to recommend qigong to young adults who fulfill diagnostic criteria for major depression or other mental disorders. Further rigorously designed RCTs adhering to accepted standards of trial methodology are required to determine more accurately the efficacy and effectiveness of qigong and its long-term effect on depressive symptoms. Particular attention should be paid to how to optimize recruitment and how to motivate relevant patients to take part in the studies.

## Figures and Tables

**Table 1:** Summary of randomized controlled trials of the effects of qigong on depressive and anxiety symptoms.

Studies	Participants (age)	Sample size (pre-/post-)	Intervention (frequency for qigong)	Control	Duration and Followup	Subjective outcome measures	Intergroup differences
Chan et al., 2012 [28]	Outpatients with clinical depression (28–62 y)	QG: 25/17 CG1: 25/17 CG2: 25/16	DMBI (90 min group practice once a week plus weekly home assignments)	CG1: CBT CG2: Waiting-list	10 wk	(1) HRSD (2) BDI-II (3) Quality of sleep	(1) *P* = 0.03 (DMBI versus waiting-list) *P* = 0.01 (CBT versus waiting-list) (2) *P* = 0.001 (DMBI versus waiting-list) *P* = 0.009 (CBT versus waiting-list) (3) *P* < 0.03 (DMBI versus Waiting-list)

Chan et al., 2011 [29]	Adults with depressive mood (25–64 y)	QG: 20/NR CG: 20/NR	DMBI (90 min group practice once a week plus weekly home assignments)	Group CBT	4 wk	BDI-II	*P* < 0.05

Cheung et al., 2005 [30]	Patients with hypertension IG: 57.2 ± 9.5 y CG: 51.2 ± 7.4 y	QG: 47/47 CG: 44/41	Goulin qigong (2 hr group practice twice a week for 4 weeks followed by once a month for 3 months plus 75 min home practice daily)	Conventional exercise	16 wk	(1) BAI (2) BDI (3) SF-36	(1) NS (2) NS (3) NS

Liu et al., 2012 [31]	Patients with type 2 diabetes mellitus (46–83 y)	QG: 44/33 Cg: 44/36	Baduanjin qigong plus health education and usual care (40 min group practice once a week plus five times home practice per week)	Health education and usual care	12 wk	(1) SDS (2) DMQLS	(1) *P* < 0.05 (2) *P* < 0.05

Ma et al., 2011 [32]	Women with perimenopausal syndrome and depression (45–55 y)	QG: 49/NR CG1: 46/NR CG2: 50/NR	Baduanjin qigong (90 min group practice once each day)	CG1: Walking CG2: Waiting-list	3 mo	CES-D	*P* < 0.01 (QG versus CG1) *P* < 0.01 (QG versus CG2)

Qiu, 2011 [33]	Outpatients with mild/moderate depression (18–60 y)	QG: 31/NR CG: 29/NR	Wuqinxi qigong plus drugs (40 min group practice once each day)	Walking plus drugs	8 wk	(1) HAMD (2) SDS (3) PSQI	(1) *P* < 0.05 (2) *P* < 0.05 (3) *P* < 0.05

Stenlund et al., 2009 [34]	Patients with burnout syndrome (25–65 y)	QG: 41/33 CG: 41/35	Qigong (style: NR) plus usual care (1 hour group practice twice a week plus home practice)	Usual care only	12 wk	(1) HADS (2) SMBQ (3) SF-36	(1) NS (2) NS (3) NS

Tsang et al., 2012 [35]	Depressed elders with chronic illness	QG: 21/19 CG: 17/15	Bajuanjin qigong (45 min group practice three sessions per week)	Newspaper reading and discussion	12 wk	(1) GDS (2) HRSD (3) CGSS	(1) *P* = 0.007 (2) NS (3) *P* = 0.025

Tsang et al., 2006 [36]	Elderly with depression (≧65 y)	QG: 56/48 CG: 41/34	Baduanjin qigong (30–45 min group practice three times a week plus 15 min home practice daily)	Newspaper reading and discussion	16 wk	(1) GDS (2) CGSS (3) PWI (4) GHQ	(1) *P* = 0.041 (2) *P* < 0.001 (3) *P* < 0.001 (4) *P* = 0.042

Tsang et al., 2003 [37]	Elderly with chronic illness and depressed mood (≧65 y)	QG: 24/NR CG: 26/NR	Bajuanjin qigong plus basic rehabilitation activities (1 hour group practice twice a week plus 30 min home practice daily)	Traditional remedial rehabilitation activities	12 wk	(1) GDS (2) WHOQOL-BREF	(1) *P* = 0.145 (2) *P* < 0.05 for physical health

Wang et al., 2010 [38]	“Subhealth problem” (n.r.)	QG: 40/NR CG: 40/NR	Wuqinxi qigong (60 min group practice five times per week)	Waiting-list	3 mo	(1) SDS, (2) SAS	(1) *P* < 0.05 (2) *P* < 0.05

Wang, 2008 [39]	Patients with type 2 diabetes mellitus and insomnia (40–70 y)	QG: 30/23 CG1: 30/26 CG2: 30/29	Baduanjin qigong (60 min group practice twice a week plus home practice daily)	CG1: mindful relaxation CG2: waiting-list	4 mo	(1) SDS (2) SAS (3) PSQI	(1) *P* = 0.036 (QG versus CG2) (2) NS (3) *P* = 0.02 (QG versus CG2)

CG: control group; QG: qigong group; NR: not reported; NS: nonsignificance.

BAI: Beck Anxiety Inventory; BDI: Beck Depression Inventory; BDI-II: Beck Depression Inventory; CBT: cognitive-behavioral therapy group; CES-D: Center for Epidemiologic Studies Depression Scale; CGSS: Chinese General Self-efficacy Scale; DMBI: Dejian mind-body intervention; DMQLS: quality of life scale for patients with type 2 diabetes mellitus; GDS: Geriatric Depression Scale; HADS: Hospital Anxiety and Depression Scale; HAMD: Hamilton depression rating scale; HRSD: Hamilton Rating Scale for Depression; PSQI: Pittsburgh Sleep Quality Index; PWI: Personal Well-Being Index; SAS: Self-Rating Anxiety Scale; SDS: Self-Rating Depression Scale; SMBQ: Shirom-Melamed Burnout Questionnaire. WHOQOL-BREF: World Health Organization Quality of Life: Abbreviated Version.

**Table 2:** Quality assessment of the included studies.

Studies	Randomization employed	Randomization methods	Inclusion and exclusion criteria	Outcome assessors blinded	Withdrawal and dropouts	Sample size estimation	Intention- to-treat analysis	Intervention described	Qualification of the trainer
Chan et al., 2012 [28]	Y	N	Y	Y	Y	N	Y	Y	Y
Chan et al., 2011 [29]	Y	Block randomization	Y	Y	N	Inappropriate*	NA	Y	Y
Cheung et al., 2005 [30]	Y	A randomization list prepared by a statistician	Y	N	Y	Y	Y	Y	Y
Liu et al., 2012 [31]	Y	Random digit number table	Y	N	Y	N	N	Y	Y
Ma et al., 2011 [32]	Y	N	Y	N	N	N	NA	Y	N
Qiu, 2011 [33]	Y	Random digit number table	Y	N	N	N	NA	Y	Y
Tsang et al., 2003 [37]	Y	Drawing of lots	Y	N	Y	Y	Y	Y	Y
Stenlund et al., 2009 [34]	Y	Computer-generated number	Y	Y	Y	N	Y	Y	Y
Tsang et al., 2012 [35]	Y	N	Y	Y	Y	Y	N	Y	Y
Tsang et al., 2006 [36]	Y	N	N	N	N	N	NA	Y	Y
Wang et al., 2010 [38]	Y	N	N	N	N	N	NA	Y	N
Wang, 2008 [39]	Y	Computer-generated number	Y	N	Y	N	N	Y	Y

N: not reported; NA: not applicable; Y: yes. *Sample size was not calculated according to power and effect size.
